# Supplementary material for: Benchmark decadal forecast skill for terrestrial water storage estimated by an elasticity framework
Source: Nat Commun. 2019 Mar 15;10:1237. doi: 10.1038/s41467-019-09245-3 (PMC6420621; doi:10.1038/s41467-019-09245-3)
Supplement: Supplementary file 1 — Supplementary Information [file 41467_2019_9245_MOESM1_ESM.pdf]

Supplementary Information for

Benchmark Decadal Forecast Skill for Terrestrial Water Storage Estimated by an Elasticity Framework

Enda Zhu[1,3], Xing Yuan[1,2*] & Andrew W. Wood[4]

[1]Key Laboratory of Regional Climate-Environment for Temperate East Asia (RCE-TEA), Institute of Atmospheric Physics, Chinese Academy of Sciences, Beijing, 100029, China

[2]School of Hydrology and Water Resources, Nanjing University of Information Science and Technology, Nanjing 210044, Jiangsu, China

[3]College of Earth and Planetary Science, University of Chinese Academy of Sciences, Beijing, 100049, China

[4]Research Applications Laboratory, NCAR, Boulder, Colorado, 80307-3000, USA

[*]*Corresponding author address:* Xing Yuan, School of Hydrology and Water Resources, Nanjing University of Information Science and Technology, Nanjing 210044, Jiangsu, China. E-mail: xyuan@nuist.edu.cn

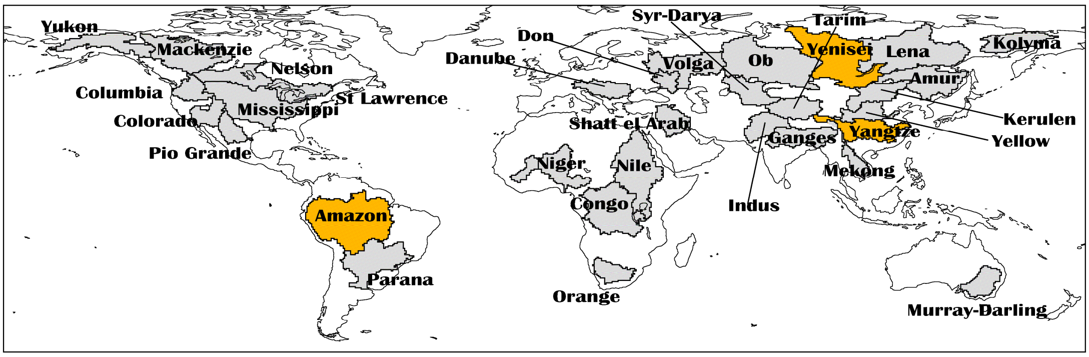

Supplementary Figure 1. Locations of 32 global major river basins analyzed in this study. Three basins with orange color were used to illustrate the terrestrial water storage (TWS) forecast skill elasticity. The map was created by using the NCAR Command Language (Version 6.3.0) [Software]. (2016). Boulder, Colorado: UCAR/NCAR/CISL/TDD. http://dx.doi.org/10.5065/D6WD3XH5.

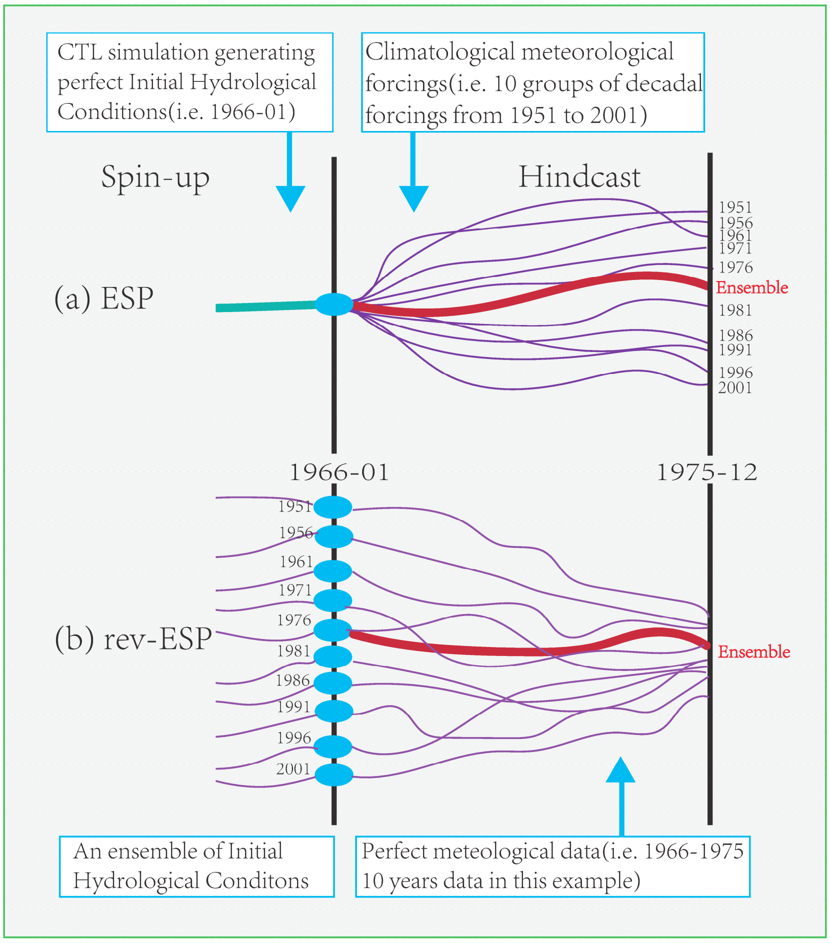

Supplementary Figure 2. Schematics of ensemble hydrological simulations. The (**a**) Ensemble Streamflow Prediction (ESP) and (**b**) reverse ESP (rev-ESP) simulations started every five years during 1951-2010. Here, 1966-01 was taken as an example to illustrate the ensembles for the ESP and rev-ESP simulations.

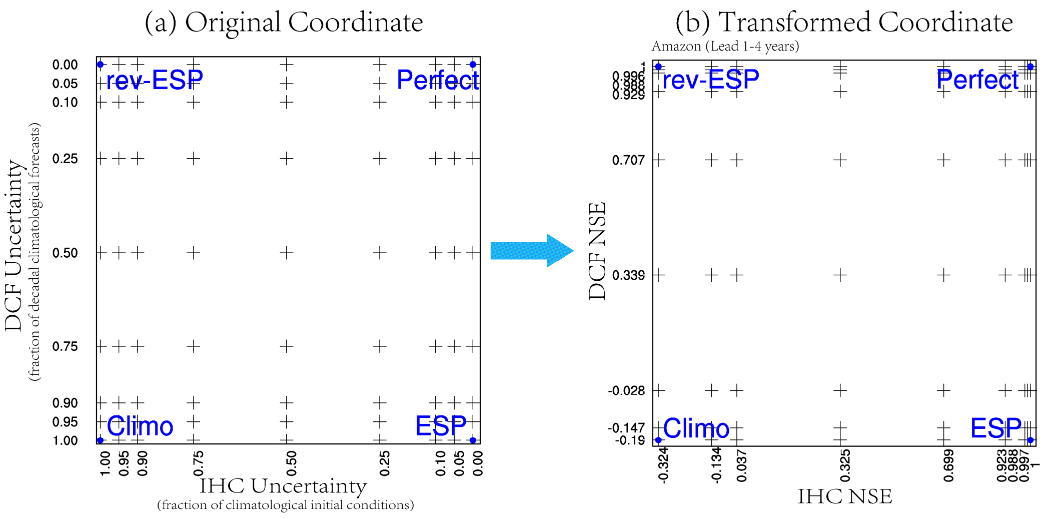

Supplementary Figure 3. Diagrams of decadal hydrological forecast skill with varying skill of initial conditions and climate forecasts. (**a**) The X and Y axes display the uncertainties (fraction of climatology) in Initial Hydrological Conditions (IHCs) and Decadal Climate Forecasts (DCFs), respectively. (**b**) The same as (a), but with transformed coordinates that measure skill in term of Nash-Sutcliff Efficiency coefficient (NSE) in IHCs and DCFs.

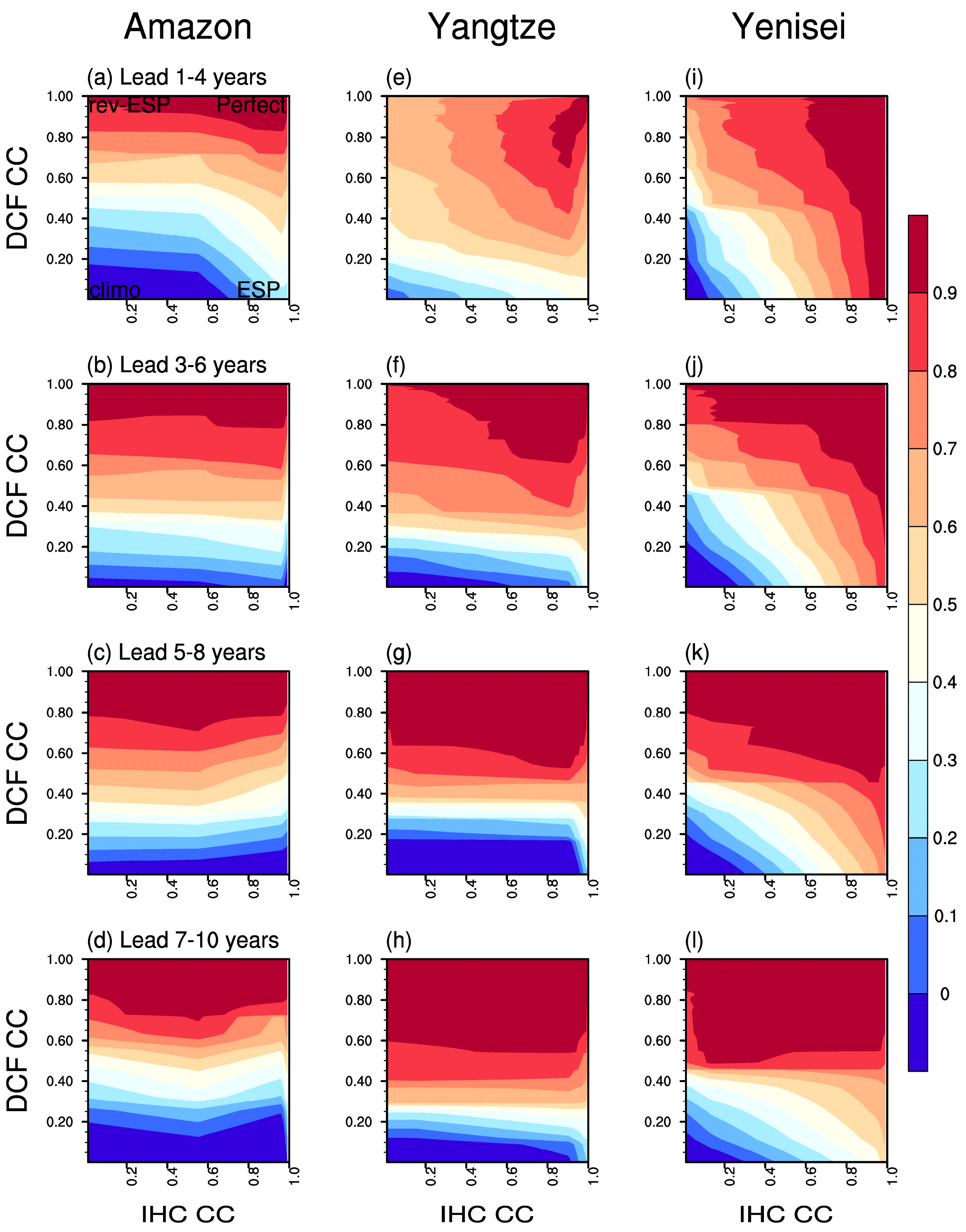

Supplementary Figure 4. The decadal hindcast skill for terrestrial water storage with varying skill of initial conditions and climate forecasts. The same as Figure 1, but correlation coefficient (CC) was used to measure predictive skill of IHCs, DCFs and TWS.

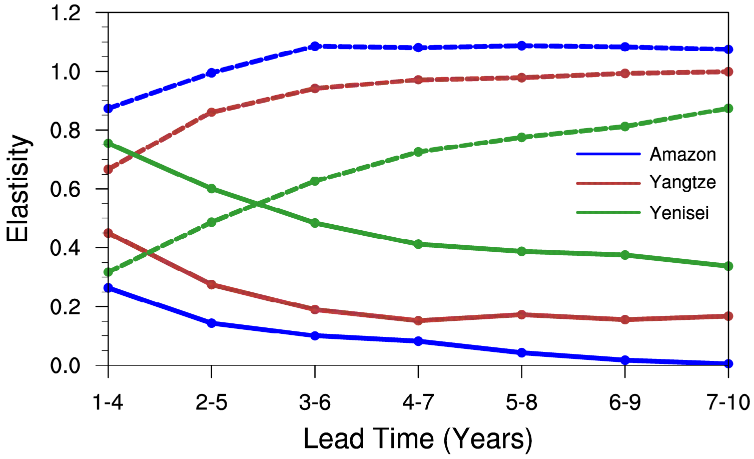

Supplementary Figure 5. Basin mean TWS hindcast skill elasticity. The results were calculated for decadal climate forecasts (DCFs; dash lines) and initial hydrological conditions (IHCs; solid lines) at different lead times.

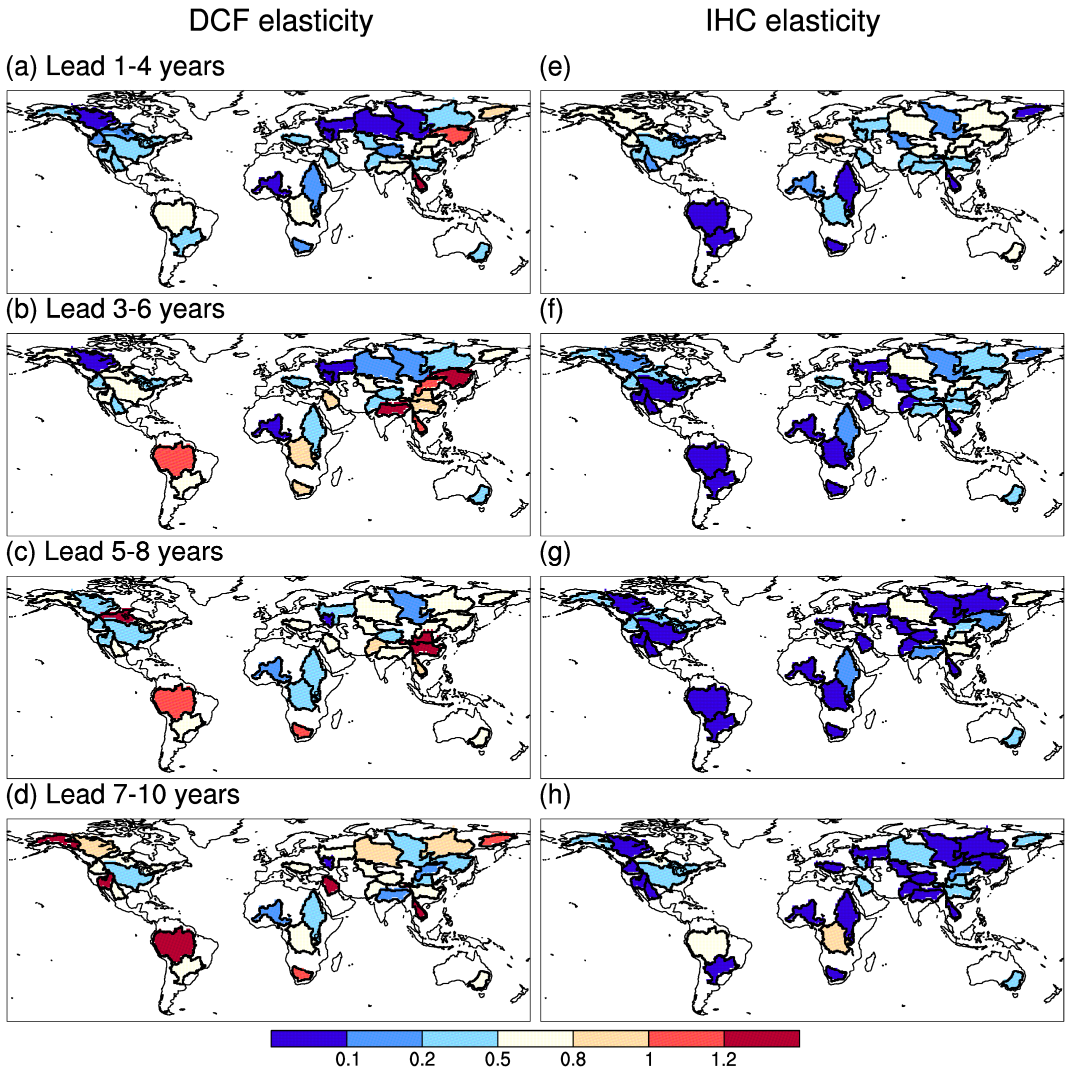

Supplementary Figure 6. Skill elasticities for climate forecasts and initial conditions. The same as Figure 2, but for DCFs and IHCs elasticities measured by correlation. Maps were created by using the NCAR Command Language (Version 6.3.0) [Software]. (2016). Boulder, Colorado: UCAR/NCAR/CISL/TDD. http://dx.doi.org/10.5065/D6WD3XH5.

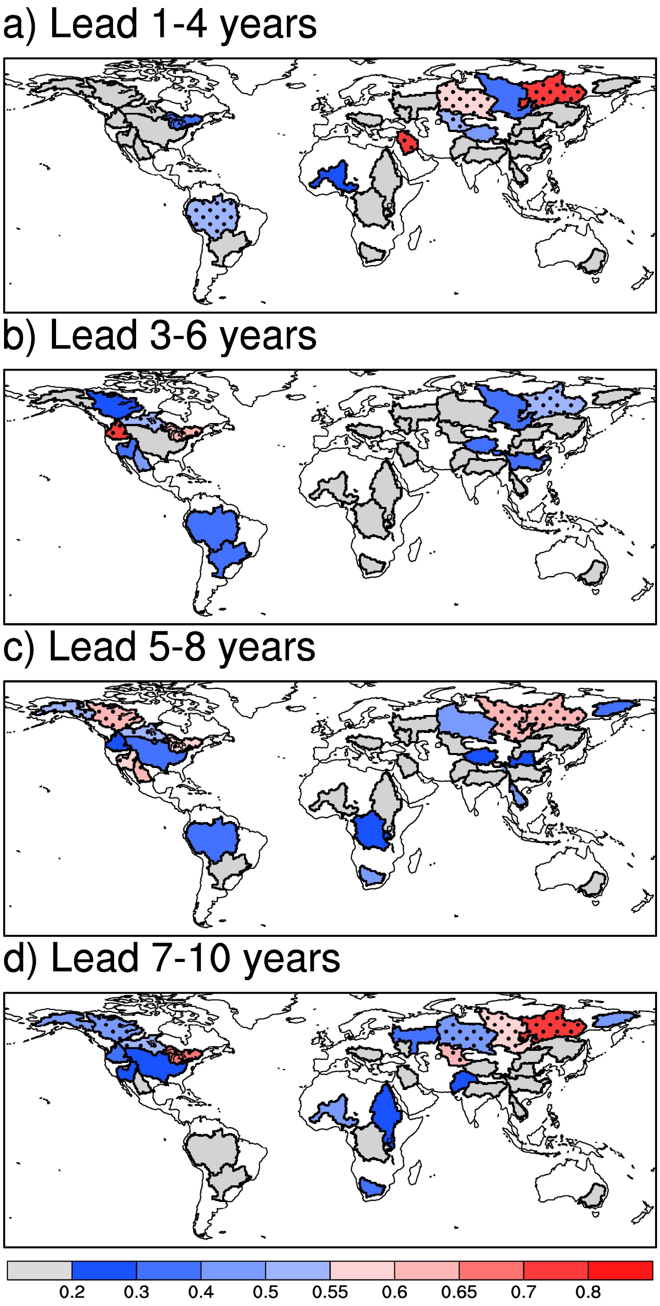

Supplementary Figure 7. Decadal hindcast skill for basin averaged precipitation. The same as Figure 3, but for CMIP5 multi-model ensemble hindcast skill of precipitation measured by correlation. The basins with significant correlations ($p<0.1$) are dotted. Maps were created by using the NCAR Command Language (Version 6.3.0) [Software]. (2016). Boulder, Colorado: UCAR/NCAR/CISL/TDD. http://dx.doi.org/10.5065/D6WD3XH5.

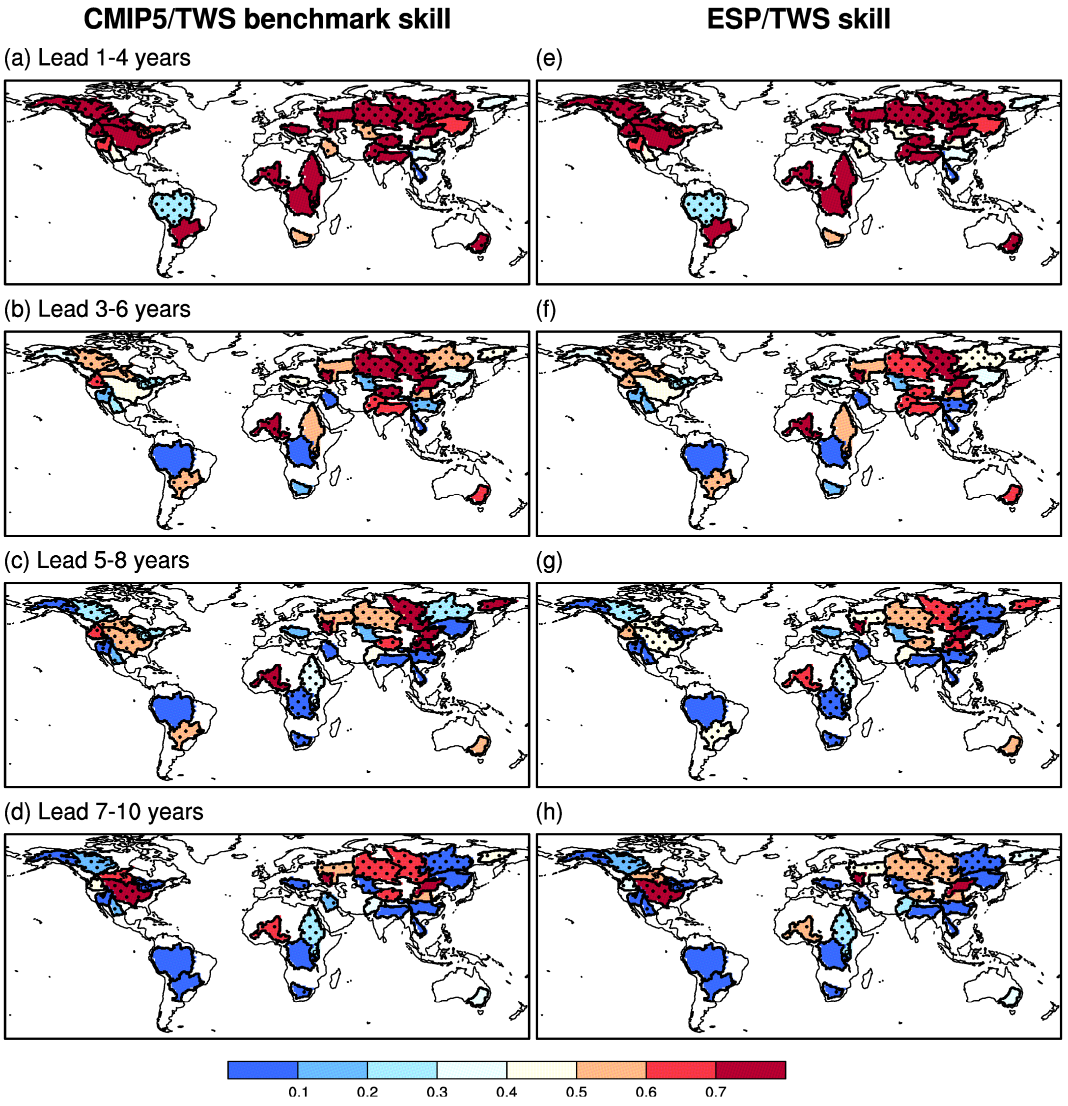

Supplementary Figure 8. Comparison between benchmark decadal hindcast skill and initial conditions-based hindcast skill. The same as Figure 4, but for benchmark skill and ESP skill measured by correlation. The regions with benchmark skill significantly ($p<0.05$) higher than ESP skill are dotted. Maps were created by using the NCAR Command Language (Version 6.3.0) [Software]. (2016). Boulder, Colorado: UCAR/NCAR/CISL/TDD. http://dx.doi.org/10.5065/D6WD3XH5.

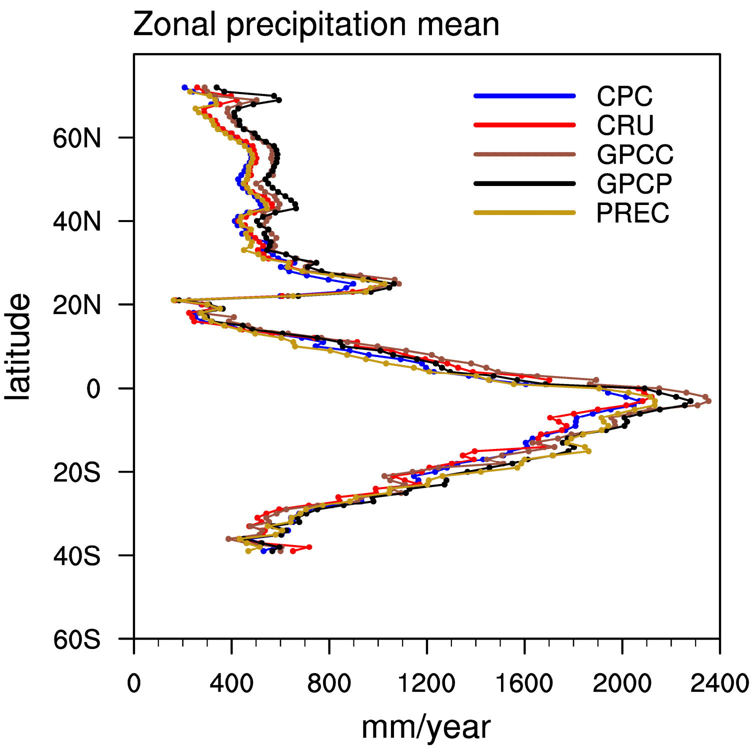

Supplementary Figure 9. Zonal mean precipitation from five datasets. Rain-gauges and satellite-based algorithms are used to produce Climate Prediction Center Merged Analysis of Precipitation (CPC) and Global Precipitation Climatology Centre (GPCP) monthly precipitation analysis product, while Precipitation Reconstruction over Land (PREC), Global Precipitation Climatology Centre (GPCC) and CRU datasets are based on a number of station observation.

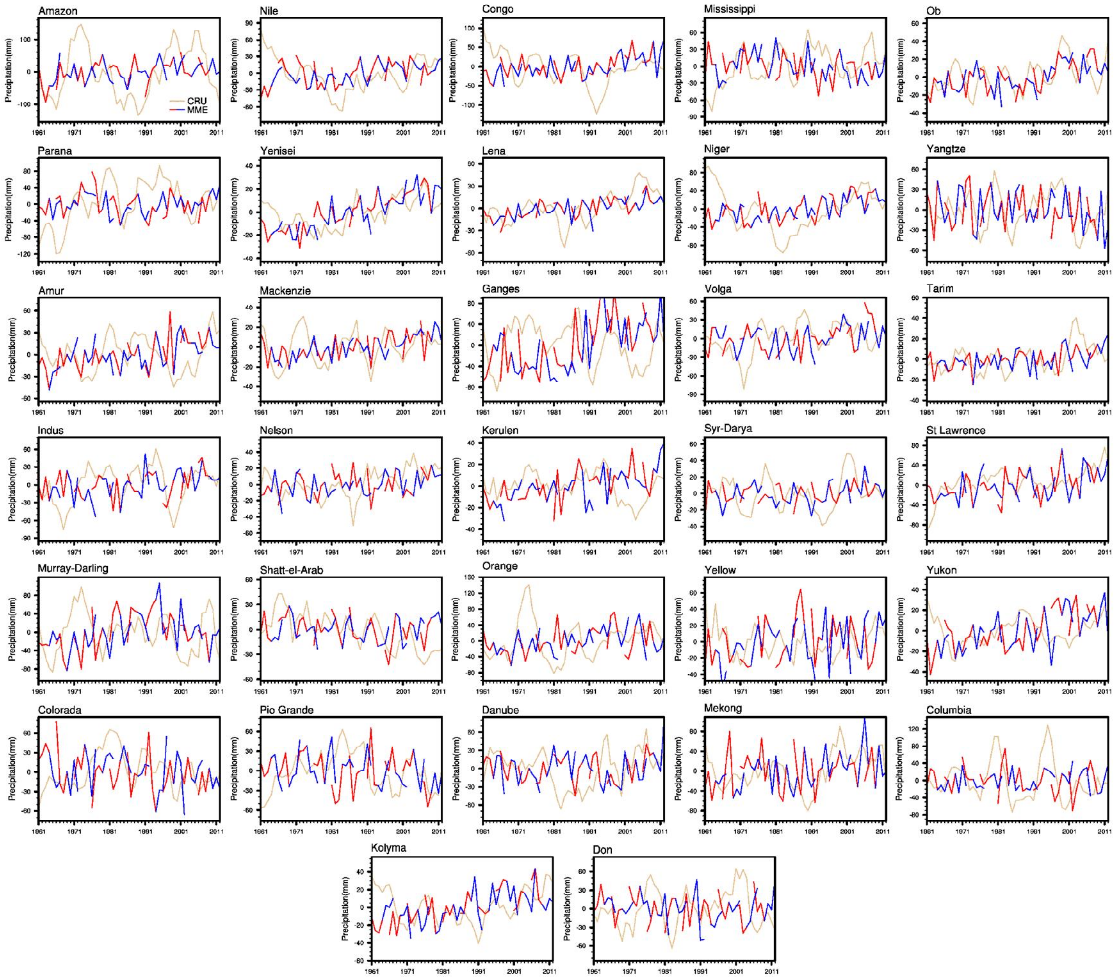

Supplementary Figure 10. Validation of decadal hindcasts of precipitation averaged over global river basins. Time series of CMIP5 multi-model ensemble 4-year mean precipitation hindcasts and CRUNCEP 4-year mean precipitation observations averaged over 32 basins. The brown lines and red-blue lines stand for CRUNCEP and CMIP5, respectively.
